# Beyond genomics: understanding exposotypes through metabolomics

**DOI:** 10.1186/s40246-018-0134-x

**Published:** 2018-01-26

**Authors:** Nicholas J. W. Rattray, Nicole C. Deziel, Joshua D. Wallach, Sajid A. Khan, Vasilis Vasiliou, John P. A. Ioannidis, Caroline H. Johnson

**Affiliations:** 1 Department of Environmental Health Sciences, Yale School of Public Health, Yale University, New Haven, CT USA; 2 Collaboration for Research Integrity and Transparency (CRIT), Yale Law School, New Haven, CT USA; 3 Center for Outcomes Research and Evaluation (CORE), Yale-New Haven Health System, New Haven, CT USA; 4 Department of Surgery, Section of Surgical Oncology, Yale University School of Medicine, New Haven, CT USA; 5 Yale Cancer Center, Yale University School of Medicine, New Haven, CT USA; 6 Stanford Prevention Research Center, Department of Medicine, Stanford University, Stanford, CA USA; 7 Department of Health Research and Policy, Stanford University, Stanford, CA USA; 8 Department of Biomedical Data Science, Stanford University, Stanford, CA USA; 9 Department of Statistics, Stanford University, Stanford, CA USA; 10 Meta-Research Innovation Center at Stanford, Stanford University, Stanford, CA USA

**Keywords:** Chemometrics, Exposome, Exposotype, Genomics, Genetic epidemiology, Metabolomics

## Abstract

**Background:**

Over the past 20 years, advances in genomic technology have enabled unparalleled access to the information contained within the human genome. However, the multiple genetic variants associated with various diseases typically account for only a small fraction of the disease risk. This may be due to the multifactorial nature of disease mechanisms, the strong impact of the environment, and the complexity of gene-environment interactions. Metabolomics is the quantification of small molecules produced by metabolic processes within a biological sample. Metabolomics datasets contain a wealth of information that reflect the disease state and are consequent to both genetic variation and environment. Thus, metabolomics is being widely adopted for epidemiologic research to identify disease risk traits. In this review, we discuss the evolution and challenges of metabolomics in epidemiologic research, particularly for assessing environmental exposures and providing insights into gene-environment interactions, and mechanism of biological impact.

**Main text:**

Metabolomics can be used to measure the complex global modulating effect that an exposure event has on an individual phenotype. Combining information derived from all levels of protein synthesis and subsequent enzymatic action on metabolite production can reveal the individual exposotype. We discuss some of the methodological and statistical challenges in dealing with this type of high-dimensional data, such as the impact of study design, analytical biases, and biological variance. We show examples of disease risk inference from metabolic traits using metabolome-wide association studies. We also evaluate how these studies may drive precision medicine approaches, and pharmacogenomics, which have up to now been inefficient. Finally, we discuss how to promote transparency and open science to improve reproducibility and credibility in metabolomics.

**Conclusions:**

Comparison of exposotypes at the human population level may help understanding how environmental exposures affect biology at the systems level to determine cause, effect, and susceptibilities. Juxtaposition and integration of genomics and metabolomics information may offer additional insights. Clinical utility of this information for single individuals and populations has yet to be routinely demonstrated, but hopefully, recent advances to improve the robustness of large-scale metabolomics will facilitate clinical translation.

## Background

The main concepts underpinning genetic epidemiology developed rapidly after the delineation of the structure of DNA. Neel and Schull provided the first description of these concepts in 1954 [1, 2]. While the original goal of genetic epidemiology was to understand the nature of population and familial genetic inheritance, it soon became evident that environmental factors and gene-environment interactions were important to consider simultaneously [3].

Currently, the study of the whole genome (genomics) has evolved into a multidisciplinary area of science with highly diverse applications [4, 5]. Improved efficiency of genome technology combined with a sharp decrease in cost has enabled genomic assessments in large study populations [6, 7] using genotyping and next-generation-sequencing (NGS) approaches [8]. Thousands of genome-wide association studies (GWAS) have tracked relationships between base-pair/gene patterns in genomic loci and hundreds of diseases or exposures [9]. However, the discovered loci from these large-scale studies still explain only the minority of presumed heritability for most phenotypes of interest [10]. Moreover, it has been established that genes alone account for the minority of disease etiology for many important illnesses such as cancer, and environmental and lifestyle influences play a critical role [11]. However, quantifying the myriad of environmental and lifestyle risk factors including diet, smoking, exposure to hazardous chemicals, and pathogenic microorganisms is challenging [12, 13]. An individual can be exposed to a complex mix of chemical and biological contaminants, with multiple sources, for varying durations across their life course. This concept has been termed the “exposome,” a framework for the collective analysis, and measurement of an individual’s exposures over their lifetime [14]. Moreover, different environmental exposures may be heavily correlated with each other or may act in concert to produce adverse effects, which makes studying them one at a time challenging for assigning causality [15]. Therefore, it is essential to find tools that can measure the cumulative impact of multiple exposures alongside their interactions with the genetic background of individuals. Several multidimensional analytical approaches have been developed, beyond genomics, that try to capture different aspects of this complexity, and their integration into environmental health is discussed in this review.

## Application of high-dimensional biology to the environmental health paradigm

Referred to as high-dimensional biology, or a multi-omics/systems-level approach, the combined analysis of data from the genome (genomics), RNA transcription (transcriptomics), proteins/peptides (proteomics), and metabolites (metabolomics) enables researchers to overlay gene information onto complementary datasets towards a more systemic understanding of diseases or other phenotypes of interest [16]. The complexity of high-dimensional datasets becomes even more convoluted when the interaction of environmental exposures is added to the system.

**Fig. 1:**
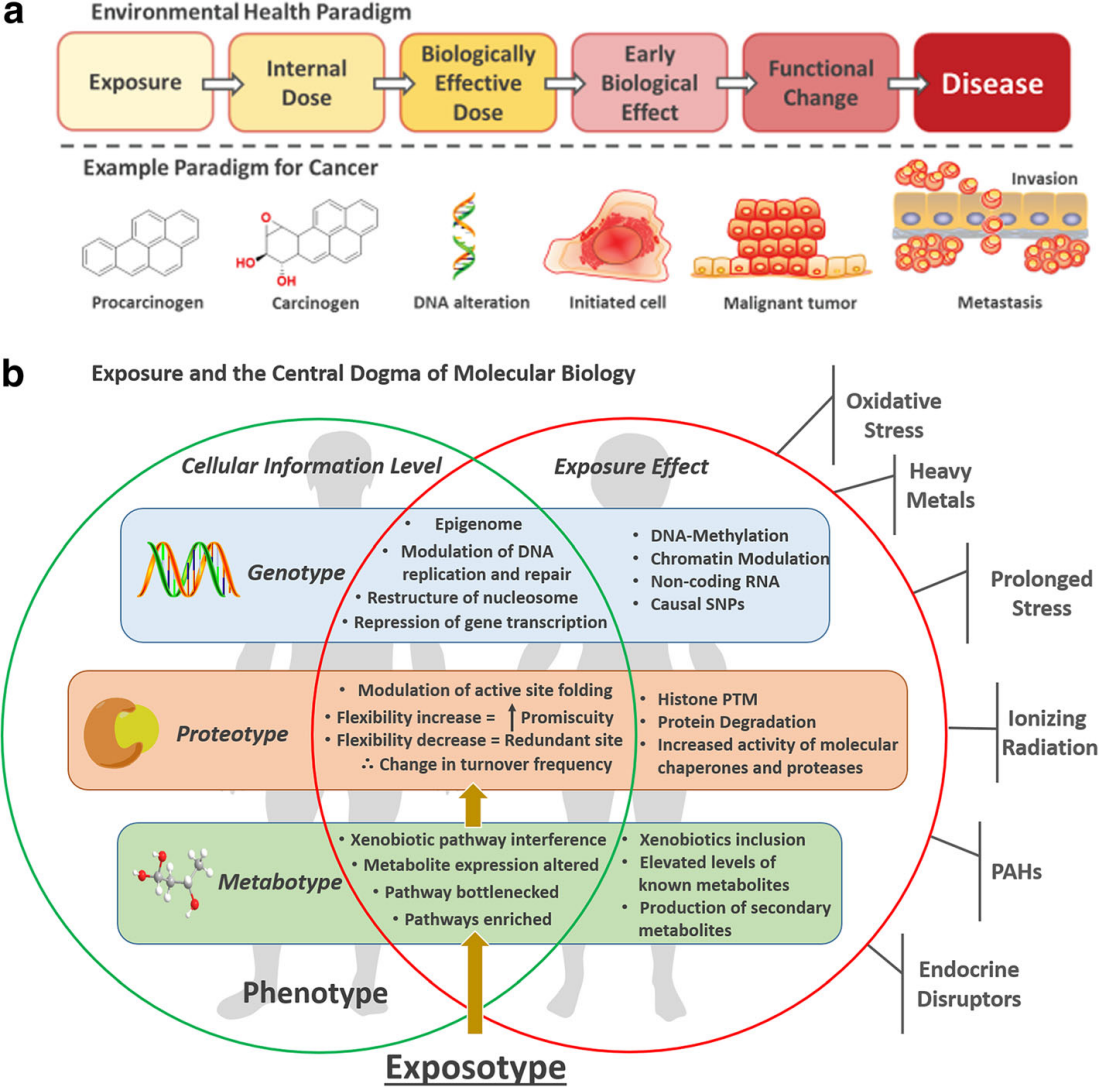
**a** Environmental health paradigm. **b** Exposure and the central dogma of molecular biology

The environmental health paradigm (Fig. 1) integrates the knowledge of exposures and environmental health sciences to gain a deeper understanding of the consequences of exposure towards expression of a disease phenotype [17]. Exposures can elicit subtle effects at different stages of gene-encoding, protein synthesis, and on circulating metabolites. Multi-omics approaches using combined data from genomics, proteomics, and metabolomics techniques can identify downstream chemical alterations contributing to the development of an exposotype, the exposure phenotype (Fig. 1), that describes the accrued biological changes within a system that has undergone a specific exposure event [18]*.* Combining information from all levels of protein synthesis and subsequent enzymatic action on metabolite production is an essential step to start comprehending the complex global modulating effect that an exposure event has on an individual phenotype. This may allow for a greater direct understanding of molecular mechanisms that underpin the route of exposure, and the effect of molecular transit on different areas of metabolism, cellular reproduction, and ultimately the resulting exposotype.

Metabolites are the substrates and products of metabolism that drive essential cellular processes such as energy production, and signal transduction [19]. Of all the molecular entities (genes, transcripts, proteins, metabolites), metabolites have the closest relationship to expressed phenotype as they are the final end-points of upstream biochemical processing. Quantitative readouts of metabolite abundance reflect both this cellular processing and xenobiotics (foreign substances such as environmental chemicals, pollutants, drugs, food additives, dyes) that are physico-chemically distinct from molecular entities that originate in the host. Xenobiotics can be processed by enzymatic machinery, and metabolomics also allows quantification of these metabolites. Therefore, metabolomics can simultaneously analyze both exogenous chemicals and their metabolites, and changes to the endogenous metabolome, to allow assessment of broadly defined exposures and their biological impact [20–23]. One such example was a recent study of occupational exposure to trichloroethylene (TCE) [24]. TCE metabolites were identified in human plasma and associated with changes to endogenous metabolites that were known to be involved in immunosuppression, hepatotoxicity, and nephrotoxicity. This allowed the investigation into how the toxic effects of TCE exposure were manifested [24]. Another study, from the EXPOsOMICS project (http://www.exposomicsproject.eu/), examined human biofluids and exhaled breath for exposure to swimming pool disinfection by-products (DBPs) and for concomitant changes to endogenous metabolites. The study revealed a possible association between DBPs and perturbations to metabolites in the tryptophan pathway [25]. However, these studies and others which have measured exposures in relation to the metabolome highlight the challenge of attempting to unravel the effect of one circumscribed exposure versus combinations of different environmental exposures on the metabolome [26, 27].

One of the major bottlenecks of metabolomics is metabolite identification. However, the expansion and development of metabolite databases have eased this issue. Tens of thousands of metabolites have been identified and uploaded onto metabolite databases such as The Human Metabolome Database (HMDB) (http://www.hmdb.ca/metabolites), which to date houses 114,113 metabolites with associated chemical, clinical, and biochemical information. HMDB also hosts four additional databases including the Toxic Exposome Database (T3DB) (http://www.t3db.ca/) which contains information on 3763 toxins [28, 29]. METLIN (https://metlin.scripps.edu), another large database containing 961,829 metabolites, recently expanded due to the integration of xenobiotics from the United States Environmental Protection Agency’s “Distributed Structure-Searchable Toxicity (DSSTox)” database [30, 31]. The Exposome-Explorer database was recently designed to contain information on biomarkers of exposure to environmental risk factors for diseases. This database has information on 692 dietary and pollutant biomarkers, and importantly concentration values measured in biospecimens, with correlation values to assess quality of the biomarkers [32]. These databases, and others that house both xenobiotics and endogenous metabolites, appear in Table 1 [33–38]. With the recent expansion of these databases to include xenobiotics, metabolomics can facilitate both biomonitoring of exposures, assessment of biological impact, and identification of exposotypes [39]. However, one potential gap in these databases still exists, the prediction of phase I and phase II biotransformed metabolites of xenobiotics which can be used as proxy biomarkers for the chemical exposure. Metabolomics has revealed numerous novel metabolites of previously well-characterized pharmaceutical drugs such as acetaminophen [40], dietary supplements [41], and the genotoxic heterocyclic amine 2-amino-1-methyl-6-phenylimidazo[4,5-b]pyridine (PhIP) [42], present in meats cooked at high temperatures. Metabolomics provides a window to identifying these new metabolites, as the biotransformed metabolite will only be present in a sample from an exposed individual. Secondly, there is typically more than one biotransformation metabolite present for each xenobiotic, which will have a similar covariance and correlation within the biological sample examined, thus making it possible to easily map out the related metabolites. One way to overcome this gap in the metabolite databases would be to have a tool housed on these databases that could automatically predict any potential biotransformations, and display the resultant important chemical information for identification. A few tools currently available for predicting phase I and II drug metabolism have been recently reviewed, along with the development of “DrugBug” which can predict xenobiotic metabolism by human gut microbiota enzymes [43]. Integration of such tools would facilitate exposome analysis.

**Table 1:** Mass spectrometry metabolite databases for identification of environmental exposures

Database name	Description	URL
Human metabolome database (HMDB)	114,113 xenobiotic and endogenous metabolites with chemical, biochemical, and clinical information.	http://www.hmdb.ca/ [33]
Toxic exposome database (T3DB)	3767 toxic compounds, targets and gene expression data, part of the HMDB suite.	http://www.t3db.ca/ [28]
METLIN	961,829 xenobiotic and endogenous metabolites with chemical information. Contains information from DSSTox.	https://metlin.scripps.edu/ [34]
Exposome-Explorer	692 dietary and pollutant biomarkers, with concentration values measured from biospecimens with intra class correlation coefficients.	http://exposome-explorer.iarc.fr/ [32]
Madison-Qingdao Metabolomics Consortium Database	20,300 xenobiotics and endogenous metabolites, with chemical information	http://mmcd.nmrfam.wisc.edu/ [35]
Drugbank	10,513 drug entries with drug target information, part of the HMDB suite	https://www.drugbank.ca/ [36]
PubChem	93,977,784 compounds, xenobiotic and endogenous metabolites but also peptides, and chemically altered macromolecules. Data is derived from hundreds of sources.	https://pubchem.ncbi.nlm.nih.gov/ [37]
CompTox Chemistry Dashboard	758,000 xenobiotics with chemical information compiled from multiple sources; PubChem, and US EPA’s DSSTox, ACToR, ToxCast, EDSP21, and CPCat.	https://comptox.epa.gov/dashboard [38]

The broad range of chemical classes that exist among the thousands of endogenous and environmentally derived metabolites contained within a biological sample has given rise to the need for analytical strategies that can separate and detect as much chemical diversity as possible from within the biological system under examination. The assessment of all metabolites present in a sample, untargeted metabolomics, is typically carried out using chromatography-based mass spectrometry and/or nuclear magnetic resonance spectroscopy, alongside bioinformatics that help understand the complex data generated [44]. Metabolomics research has undergone significant refocus over the past few years due to the improvements made in bioanalytical protocols and an evident shift towards the development of new chemoinformatic and bioinformatic tools [45]. These tools are designed to improve metabolite identification, particularly for microbial metabolites, and biological interpretation, which remain a major challenge for the field. For example, the mass spectrometry data generated in a metabolomics study have a high degree of degeneracy where the same metabolite can be represented as multiple signals [46]. Tools such as CAMERA [47], RAMClust [48], and “Credentialing” [49] have helped overcome this problem and improve peak annotation. Other notable tools include CSI:FingerID [50] which predicts the fragmentation of metabolites using an in silico method, thus aiding in metabolite identification, and “integrated-omics” housed on XCMSOnline [51] (http://xcmsonline.scripps.edu/) which aids in both metabolite identification and biological interpretation. Excellent reviews on the technological advancements in this area can be found elsewhere [52–54]; in addition, an extensive list of all current metabolomics software and data analysis resources is available [55, 56]. For population-level studies, the application of metabolomics for the analysis of thousands of samples has been optimized and demonstrated [57, 58], but the field could still benefit from decades’ worth of research and lessons learning in genetic epidemiology related to study design, statistical analyses, and reproducibility in large-scale population consortia.

## Methodological challenges and considerations

Relevant and a priori formulated research questions and rigorous study designs and methods lay the foundation to perform a potentially successful piece of population-based research, after which replication is essential to confirm any associations, and to avoid the dissemination of potentially false research claims [59–61]. Prospective cohort studies follow a predefined population over time, capturing exposure information prior to occurrence of health events. This study design accommodates the appropriate temporal relationship between exposure and outcome, allows for testing of multiple risk factors and health outcomes, and permits collection of multiple pre-clinical biological specimens throughout the follow-up period. Although this is ideal from a metabolomics perspective, this study design often requires long follow-up durations and great expense. Case-control studies can be more efficient, and less expensive ways to test associations, but they lack the temporality criterion for causality, and metabolic profiles may be influenced by disease status. The use of nested case-control studies offers an efficient approach with the appropriate temporality between exposure and outcome. “Meet-in-the-middle” approaches, which involve linking intermediate biomarkers to both the exposure and outcome within cohort and nested case-control studies, are gaining popularity for their ability to reveal important linkages along the exposure-outcome pathway [62, 63].

While systems-level approaches hold great promise, they also pose challenges in the analysis of high-dimensional, complex data structure. The use of appropriate statistical tests within genomics, metabolomics, and epidemiology is dictated by the study design and the number of dimensions of data under investigation, with the application of univariate or multivariate techniques being applied to low-dimensional and high-dimensional datasets, respectively. Incorrect analytical decisions and interpretations that are made when conducting a study are a direct threat to reproducibility [64]. Table 2 [65–87] provides a list of some of the most commonly used statistical methods and tests in the interface of epidemiology, genetics, and metabolomics.

**Table 2:** Common statistical methods and tests used in epidemiology, genetics, and metabolomics, with reference link to descriptive articles on appropriate general use

Class of test	Type of test	Application/description	Refs
Descriptive	Mean Median Mode	The simplest of tests used to describe basic features within data.	Covered in all general statistical textbooks and used in most if not all scientific disciplines. [67–69]
	Range, variance, SD	Describe spreads of data within a population	
Inferential	*z* test, *t* test, chi-square	Predicts/infers an observed mean, frequency, or proportion to a predetermined value, respectively.	
	ANOVA	Parametric method that tests the hypothesis that the means of two or more populations are equal. Frequently used to compare variance among groups relative to variance within groups	
	Kruskal-Wallis	Non-parametric method to rank statistical significant differences between two or more groups of an independent variable on a continuous/ordinal variable	
Scaling	Centering, auto, pareto, log, MD	Data pretreatment methods aim at reducing biological and analytical bias	[70, 71]
Principal component	PCA	Unsupervised dimensional reduction procedure used to explain the maximum variance within complex datasets.	[72–74]
	Multiblock PCA	PCA extension designed to find the underlying relationships between sets of related data	[65, 66, 75]
	ANOVA-PCA	Uses PC dimensional reduction to determines the effect of the experimental factors on multiple dependent variables	[65, 76]
	PC-DFA	Supervised test that summarizes the differentiation between groups while overlooking within-group variation.	[65, 77, 78]
Regression	Linear	Summarizes and quantifies the relationship between two continuous variables	[72, 79]
	PLS	Used to predict a set of dependent variables from a large set of independent variables	[73, 77, 80–82]
	O-PLS	orthogonal signal correction on PLS that maximizes the explained covariance on the first latent variable	[77, 81, 83]
	PLS-R	Combination of the predictive power of regression alongside the ability to deal with high dimensionality and multicollinearity of variables.	[77, 84]
	PLS-DA	Supervised approach to prediction on discrete variables	[77, 79, 83]
	LASSO	Parsimonious approach to variable selection and regularization in order to enhance interpretability and reduce noise	[79, 80, 85–87]
	Elastic net	Variable reduction approach where strongly correlated predictors coalesce in or out of the model together	[79, 80, 85, 87, 167]

Definitions: *SD* standard deviation, *MD* median, *PCA* principal component analysis, *ANOVA* analysis of variance, *PC-DFA* principal component discriminant function analysis, *PLS* partial least squares (also known as projection of latent structures), *O-PLS* orthogonal PLS, *PLS-R* PLS regression, *LASSO* least absolute shrinkage and selection operator

Many analyses in metabolomics involve the use of null hypothesis significance testing (NHST) and the reporting of *p* values. The *p* value, one of the most misused statistics in science [88], has not escaped the focus of members of the fields of epidemiology [89], metabolomics [90], and general biomedicine [91]. Poor application has contributed to the irreproducible nature of many studies, so much that the American Statistical Association felt moved to release a statement highlighting six underlying principles to dictate the proper use and interpretation of the *p* value [92, 93]. One should examine in each application whether NHST is best suited as an inferential tool or whether alternative approaches, such as the use of Bayesian methods or false discovery rates (FDR), are preferable [90, 94–96]. If *p* values are still used in multidimensional experiments, proper correction for multiplicity is important. There are numerous methods for accommodating family-wise error rates [90]. There are also some standard thresholds that can be used in specific settings, e.g., genome-wide significance *p* < 5 × 10^− 8^ for genome-wide analyses. Some multiplicity corrections are more conservative than others; for instance, the Bonferroni correction (dividing the *p* value threshold required for significance by the number of tests performed) may be too conservative [97]. FDR and variants of FDR may be better suited [96] and can accommodate correlation structures between the multiple tested variables [98, 99].

Several methods are available that can help reduce complexity, detect trends, and generate predictive models within multidimensional datasets (Table 2) such as those generated by NGS and mass spectrometry when target genes or metabolites are not known. Unsupervised methods such as principal component analysis (PCA) provide an initial step to help reduce the complexity and indicate variables of interest by determining discriminant features linked to the “loadings” of different clusters. These loadings can be considered as the impact that a certain variable has on measured variance, so a high-level loading value displays a strong influence on clustered groups [100]. There also exist several extensions of the PCA architecture such as multiblock PCA, consensus PCA, or ANOVA-PCA that enable the user to control for underlying influential factors within datasets such as the intra-patient variability or other experimental confounders [65]. These approaches have been used for metabolomics and genetics analyses and also lend themselves to other cross-validation methods [66]. Supervised methods apply grouping stratification to the data based on some already known outcome variable(s). They aim to develop models that can accurately predict the correct grouping based on the input and identify genes, metabolites, or other statistical associations that underlie the grouping. The most commonly used methods are variants of regression tools (Table 2). Regression modeling can identify associations relevant to the disease [101], can predict association within gene expression patterns [102], and in metabolomics [103] can generate sample classification. However, as these tests are supervised, one of the issues with multivariate regression is that it tends to over-fit the data. Therefore, cross-validation (in the same dataset) and external validation (in additional datasets) are essential.

Perhaps, the biggest challenge yet for exposome researchers is integration of the multiple types of data generated from systems-level analyses and assessing the role of one versus multiple exposures on the phenotype. Currently, there are platforms that enable biochemical pathway analysis and integration of systems-level data, and these platforms can identify pathways and networks that are related to a known exposure or health outcome (such as disease). Dissection of pathways may help direct mechanistic studies into causality. The most useful to date for untargeted metabolomics data is “mummichog,” which uses computational algorithms to predict metabolic pathway effects directly from spectral feature tables without prior identification of metabolites [104]. Mummichog was recently integrated onto the XCMSOnline platform, with an added function to upload transcriptomic and proteomic data, for integrated pathway analysis [51]. Other notable software includes MarVis-Pathway [105], InCroMAP [106], GAM [107], and MetaCore™ (Thomson Reuters Corporation, Toronto, Canada) that can integrate multiple types of systems-level data for pathway interrogation. Combining this type of data with multiple measurements of xenobiotics has not yet been demonstrated, but tools are under development. Up to now, studies have primarily assessed the effect of individual exposures and have combined multiple systems-level approaches to assess biological response (i.e., benzene exposure and toxicity, susceptibility genes, mRNA and DNA methylation) [108]. Phenome data has also been integrated into studies to account for population variability and reduce false positives [22]. A recent example, from the analysis of preterm birth in the Rhea mother-child cohort study, selected those metabolites that had significant association with birth outcomes in logistic regression models and significant correlation coefficients with metabolic syndrome traits to construct odds ratios (BMI, blood pressure, blood glucose) [109]. Moreover, new tools are being specifically designed with the exposome in mind; xMWAS can integrate metabolomics data with that derived from the transcriptome [110], microbiome [111], and cytokine [112] and can be used for genome, epigenome, proteome, and other integrated omics analyses. However, modeling the effect of combined exposures is extremely complex. Co-exposures can be linked and cause an additive effect on the biological outcome, but it is not possible to know beforehand which combinations of exposures may have the largest biological effect. A recent novel method was developed that first estimates the correlation between pairs of exposures, then groups the highly correlated exposures by unsupervised machine learning [26], and identifies co-occurring exposure networks. This technique reduces the total number of combinations of exposures to “prevalent co-occurring combinations”; however, integration with other systems-level data still remains very complex. The additional challenges associated with integrating exposome data with metabolomics, genomics, and proteomics have been recently reviewed [27] and were also highlighted in a recent symposium report [113].

## Analytical bias and biological variance in metabolomics analyses for epidemiologic studies

Metabolomics analyses in epidemiologic studies require additional consideration of sources of variability beyond traditional epidemiologic studies. There are a very large number of chemical features that can be detected by current highly sensitive mass spectrometers, and differences in metabolite recovery may arise from biological samples that are not collected under identical protocols. Additional batch variation can be introduced when handling large sample numbers [114], due to contaminant build-up and sample degradation [115].

**Fig. 2:**
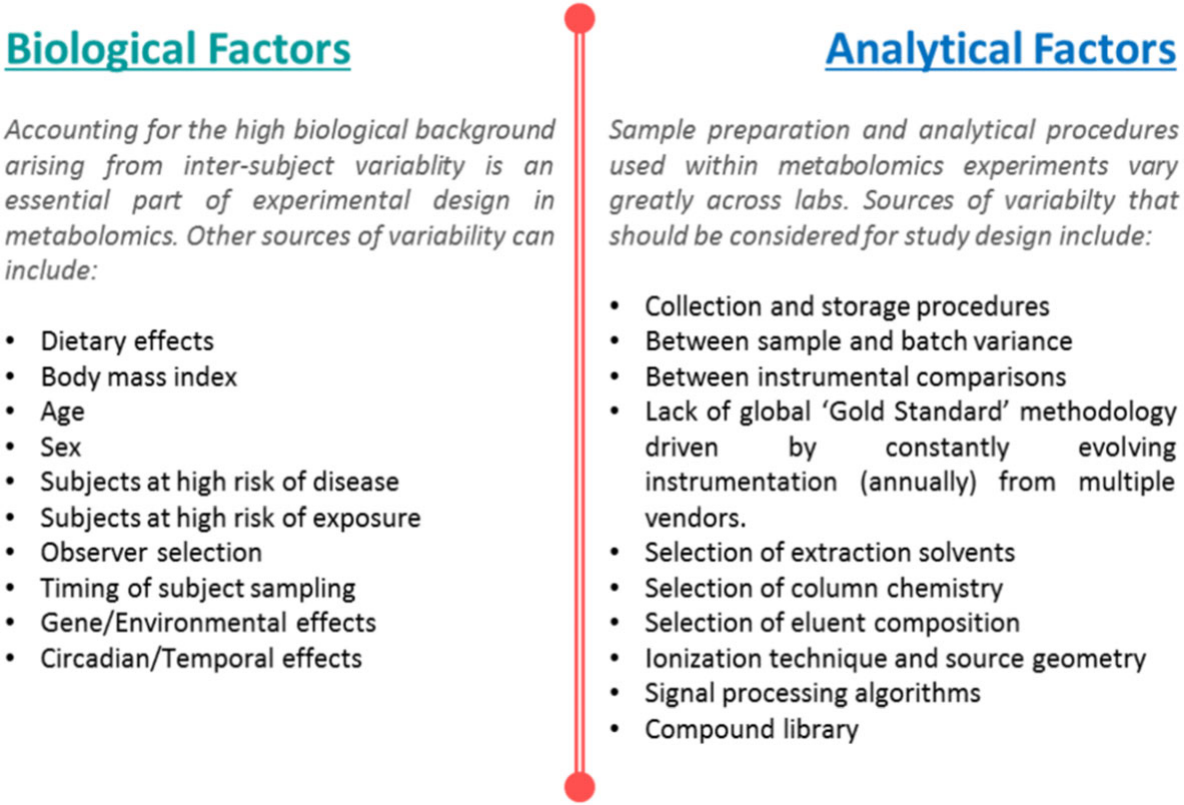
The biological and analytical aspects of bias and variance that can lead to a tendency towards erroneous results in both untargeted and targeted metabolomics

Analytical bias in genomics and metabolomics can arise from practical laboratory aspects that, by their nature, favor the preselection of one type of variable (single nucleotide polymorphism (SNP) or chemical) over another. This is particularly evident when performing “untargeted” analyses in which the researcher is looking to maximize chemical coverage with a technology that cannot cover the full chemical space. With currently over 24 million SNPs having been documented within the human genome [116], the technology within SNP microarray chips has yet to catch up to this depth of coverage. The same issues are also present within metabolomics as no single technology can analyze the thousands of different metabolites within a sample. Therefore, pre-selecting approaches are commonly applied, be it using a gene-expression chip predefined for a subset of SNPs [117–120] or untargeted chromatography methods for metabolomics with a restricted spectrum of which metabolites can be captured [121]. These analytical biases are described in Fig. 2, but include the type of metabolite extraction method and column chemistry, which can enhance the analysis of some chemical functional groups and classes over others. For example, reversed-phase liquid chromatography (RPLC) can effectively analyze non-polar compounds such as lipids, carnitines, and bile acids, whereas hydrophilic interaction liquid chromatography (HILIC) is more suitable for the analysis of polar metabolites such as nucleotides, sugars, and amino acids. The two column chemistries have an analytical overlap of only 34%; thus, both column chemistries are needed if one wishes to obtain a relative quantification of the broadest chemical classes from a sample [122]. All types of study design need to consider inherent biological intra-individual variability as a potential source of variation (Fig. 2) as well as a source of discriminatory features. In addition to understanding and addressing potential methodological challenges and various sources of biases, open science practices are necessary to support the subsequent verification of research and use of the obtained data and results in subsequent secondary analyses and meta-analyses.

## Moving from genome-wide association studies (GWAS) to metabolome-wide association studies (MWAS)

One of the most-used study approaches in big data genome research, first demonstrated in 2005, is GWAS [123]. This technique examines genome-wide sets of genetic variants in samples of individuals to determine if any variants are associated with a trait and help pinpoint genes that may contribute to a person’s risk for a certain disease or other phenotype of interest. GWAS can be described as an untargeted and sometimes a hypothesis-generating approach to associate genetic variants with specific phenotypes. GWAS and consortia-based meta-analyses have been conducted with increasing sample size [124], allowing for improved power [125] to detect genome-wide significant signals for what are typically very small effect sizes. Due to the analytical uniformity of sequencing, this is one area where genomic research has advanced more quickly than metabolomics.

Most of the early untargeted metabolomics experiments have had limited sample sizes (*n* = 10–100) often a result of technological, run-time, and statistical limitations. Given the large number of metabolic features that are typically generated by untargeted metabolomics (typically 1000s for liquid chromatography mass spectrometry), using such small sample sizes has led to over-fitting of data and spurious results [100]. Moreover, the highly collinear nature of metabolomics multivariate data [67] have not generally been properly factored in performing a priori power and sample size calculations, and there is no widely accepted method for sample size determination in metabolomics. In the absence of specific metabolic target hypothesis, one can use a data driven sample size determination (DSD) algorithm [126] where sample size estimation depends on the purpose of the study: whether it aims to find at least one statistically significant variation (biomarker discovery) or a maximum of statistically significant variations (metabolic exploration). Alternatively, one may adapt methods that have been developed for use with microarray gene expression(s) [127–129]. One common problem is that there is often high correlation between variables in one dataset, and in addition, not all variables have the same power. However, new more promising approaches have been generated using multivariate simulation to deal with this type of data structure [130].

Predictive power increases with sample size, and the current application of metabolomics to larger longitudinal cohort studies (*n* > 1000) is helping to give access to broader population data that can be linked to specific exposure such as alcohol [131, 132]. These types of studies are needed to improve biomarker discovery and inference of molecular mechanisms. Key issues continuously arise in the application of metabolomics to human subjects which can be overcome by putting metabolomics into epidemiological context. Common problems include causal and mechanistic claims based on differences between groups that have low numbers of individuals, lack of longitudinal data to avoid the possibility of reverse causation (a health outcome influencing pharmacokinetics and metabolite concentrations), limited information on lifestyle, socioeconomic and other influences, and the lack of multiple statistical tests and biological replication [133]. As metabolomics is incorporated into more population-level studies, it may be possible to more reliably model potential associations of metabolic profiles with phenotypes. The goal is to stratify metabolic data over exposure event data and ultimately determine the related disease risk. Confounding associations may still distort results and lead to erroneous conclusions. Yet it is more readily possible, with larger study numbers, and longitudinal testing, to control confounding by matching samples in to related sub-groups such as age, sex, or level-of-exposure.

Metabolome-wide association studies (MWAS) were first described in 2008 as the capture of “environmental and genomic influences to investigate the connections between phenotype variation and disease risk factors” [134, 135], thus helping reveal the complex gene-environment interactions on disease outcome. The method differs from conventional metabolomics in that high-throughput metabolomics is applied to large-scale epidemiologic studies at the population level and uses specialized algorithms to maximize the identification of biomarkers of disease risk [57]; for example, a recent algorithm was developed to correct for multiple testing using a permutation-based method to derive a metabolome-wide significance level controlling the family-wise error rate [136]. Initial studies showed that using high-throughput metabolomics, MWAS can be carried out on large population cohorts to provide individual metabolic phenotypes (metabotypes), and metabolic biomarkers correlated to exposures [137], and/or biological outcomes [138]. The proof-of-principle study used to coin the term MWAS identified discriminatory biomarkers of blood pressure and cardiovascular risk in 4630 individuals [138]. These types of studies may point to otherwise unknown features of the disease etiology or pathophysiology, which may be used to lead further mechanistic studies and potentially new avenues for therapeutic design, although the complexity of mechanisms makes such translation to therapeutic discovery very difficult. Comparison of metabotypes at the human population level can identify a signature of metabolites statistically correlated to disease risk and/or an exposure. Recent studies have shown the application of MWAS to identify metabolites correlated with cardiovascular events in a dietary intervention trial [139]. In another study, trimethylamine *N*-oxide (TMAO) was identified as a biomarker predictive of cardiovascular disease risk [140, 141] and was also shown to be involved in the production of atherosclerotic plaques. This discovery has resulted in a clinical test for TMAO, Cleveland HeartLab, and is the first to provide this blood test, and therapeutics are currently being designed to inhibit TMAO production as well as recommendations for dietary changes. Another application is to identify the enrichment of metabolites within specific biochemical pathways [142] to aid in the identification of genes and proteins/enzymes that may be related to the mechanism of disease. This method has gained traction within drug evaluation studies [143] trying to obtain more comprehensive understanding of individual responses to drug therapy [144, 145]. This application may be particularly useful for the design of immunotherapeutics where metabolites have been shown to modulate autoimmunity and can be targeted to improve the efficacy of these drugs [146, 147]. However, it should be acknowledged that therapeutic discovery or improvement in therapeutic management with known interventions has not yet been accomplished using metabolomics data; however, recent development in metabolomics technologies in both the bioanalytical and chemometric components is markedly improving, and thus, there is optimism for clinical translation as well.

## Transparency, reproducibility, and open science

There is growing recognition of the need for improved transparency, reproducibility, and replication in the biomedical literature [64, 91, 148, 149]. With respect to multidimensional, big data analyses, transparency can be improved with the sharing of data, protocols, and analytical codes. Furthermore, the number of metabolomics studies that investigate reproducibility across multiple research centers are few in number, and ongoing interlaboratory efforts have struggled to generate metabolite data that is both accurate and reproducible across different labs [150]. Replication has been accepted as a sine qua non in certain disciplines, such as human genome epidemiology [149], and the same should apply across all multidimensional fields using big data. However, the research community is aware of this issue, and groups are convening to provide solutions to address this problem. For example, the European Centre for Ecotoxicology and Toxicology of Chemicals have provided a framework to facilitate the regulatory applicability and use of big data in chemical risk assessment [151, 152].

It is also important to protect inferences from data dredging/p-hacking (mining datasets prior to specifying a causal hypothesis), and unaccounted multiple comparisons in complex datasets that can lead to the inflation of false-positive rates. Therefore, to improve the reproducibility of metabolomics, it is necessary to understand certain methodological and statistical challenges, to protect against analytical biases and biological variance, and to promote transparency and open science. These open science practices, which include “the process of making the content and process of producing evidence and claims transparent and accessible to other researchers” [64], can increase the credibility of research. For metabolomics in particular, both raw and metadata are essential to facilitate reproducibility, secondary analyses, and the synthesis of evidence by external metabolomics researchers [153]. Several measures can support the transparency and reproducibility of metabolomics. For maximal impact, the whole metabolomics research community should adopt and adhere to standards that promote the uniform preparation of study results. The metabolomics standards initiative (MSI), which was conceived in 2005 by the Metabolomics Society, highlights a range of minimum reporting standards covering biological [154], chemical [155], analytical, and data reporting methods [156] within the metabolomics experimental pipeline. However, ideally, metabolomics funders, reviewers, editors, and journals should require researchers to share their protocols, raw data, and analytical code. Broadly speaking, this does not happen (the Springer Journal *Metabolomics* (https://link.springer.com/journal/11306) and MDPI journal *Metabolites* (http://www.mdpi.com/journal/metabolites) being notable exceptions in which MSI compliance is asked for from authors and assessed by reviewers). Currently, most journals leave the suitability of metabolite submission data to reviewer and editor discretion.

Support is also beginning to appear from some funding bodies to help improve the reliability and efficiency of metabolomics. For example, the Data Repository and Coordination Center, which is part of the United States National Institutes of Health (NIH) Common Fund’s Metabolomics Program, has created the Metabolomics Data Repository. All NIH Common Fund Metabolomics Program supported research projects which create metabolomics data as part of the funded research are required to submit all raw data (e.g., spectrometric, spectrographic, and chromatographic data) and metadata (e.g., details on how samples were obtained and the analytical methods that were used) to the repository [157]. In addition, the European Union funded data repository MetaboLights (http://www.ebi.ac.uk/metabolights/) has already assembled data from 317 metabolomics studies as of December 2017. Common data submission formats, such as *mzML/mzXML* for mass spectrometry, *nmrML* for NMR data, and ISA-Tab format for metadata, have helped to unify this process [158, 159]. But the research community must be careful to not generate an excess of unconnected data repositories. Multiple and potentially overlapping repositories could confuse researchers as to where they should submit their data and therefor limit the chance of uniform acceptance and adoption of standards. To this end, the COSMOS project (COordination of Standards in MetabOlomicS—http://www.cosmos-fp7.eu/) has been designed to address the challenges of e-infrastructure diversity in metabolomics by developing an interface that globally links community projects and output.

The predominant reason behind the lack of data sharing in metabolomics is the complexity and lack of standardization in the data generated. For research areas such as genomics, transcriptomics, and, to a lesser extent, proteomics, the chemistry of the molecules under detection is highly symmetrical. Regardless of nucleobase-pair connectivity, DNA and RNA constructs can be detected and typed using highly reproducible sequencing chips that can work in a high-throughput manner. The sheer range of molecular chemistries available within the human metabolome demand a multitude of separation strategies when mass spectrometry is used as the detection technology. Consequently, different research groups align their experimental pipelines to one of the many instrument vendors (often dictated by geography and cost) leading to a multitude of protocols that cover all aspects of experimentation. Just within the confines of liquid chromatography mass spectrometry-based metabolomics, 84% use open source software and/or commercial software from instrument vendors, and within the open source software group, the majority use XCMS, and a smaller percentage use MZmine and MZmine 2. Therefore, variability in just the data processing limits integration of the MSI. One way to enable standardized data processing and biostatistics is to encourage the use of a universal workflow platform such as Galaxy (https://galaxyproject.org) [160]. In addition, the use of a standard reference material that can normalize and compare the detection levels from different instruments would be of value. A concerted effort is still needed by the community to enable broader reproducibility [161]. The lack of standardization and reporting is preventing the validation of metabolomics research [162].

## Conclusions

Human populations are exposed to a complex mix of chemicals and toxicants, from multiple sources, for varying durations. These exposures are affecting the health of the global population dramatically, for example, over seven million premature deaths annually linked to air pollution exposure alone [163]. It is vital that a more comprehensive understanding of how these environmental exposures affect biology at the systems level to determine cause, effect, and susceptibilities. In doing so, a compound specific “exposotype” can be developed that accounts for the totality of the multileveled downstream biological changes that an individual exposure event produces [18]. To better understand these effects, metabolomics can be used to develop not only metabolic biomarkers of exposure but can also be used to build metabolic models that identify upstream genetic and enzymatic changes. This may complement GWAS studies as knowledge of a potential enzymatic mutation can narrows down the DNA search space needed to identify relevant SNPs linked to the exposure [144, 145].

In-depth biological data generated by metabolomics can be used to enhance exposure studies by supplying information not only on directly affected metabolic pathways but also on off-target metabolic effects. The value of metabolomics to identify gene-environment interactions lends itself to the study of the exposome and will be the most complex and important integration of metabolomics to date. Further characterization of gene variants associated with those metabolic pathways could help forecast disease prevalence by either using pre-diagnostic metabolic signatures (collections of metabolites that change prior to disease onset) and genetic risk data. Therefore, preventive measures may be tailored specifically for those individuals. The combination of metabolomics with genomics offers one tool that may prove helpful towards materializing precision medicine. Success in precision medicine has been difficult to achieve [164], but the recent US Food and Drug Administration approval of pembrolizumab, a “tumor-agnostic” therapeutic which targets any solid tumor with a specific genetic feature, shows that the field is starting to head in that direction [165]. Given recent evidence that non-genomic influences such as the microbiome can influence therapeutic response, metabolomics may be used in this context to identify factors that are related to non-responders and responders [166].

However, some of the caveats that still exist within conventional metabolomics and population studies are still present, such as accurate identification of new metabolites, controlling for multiple levels of confounders, and the integration of different forms of data from different analytical platforms. Further advancement can be made by routine application of appropriate statistical tools to metabolomics as well as the adoption and promotion of transparent and reproducible research practices. Reproducible, transparent advances may then be examined for their impact in changing outcomes in single patients and at the population level to judge their utility.

## Abbreviations

FDR: False discovery rate
GWAS: Genome-wide association studies
MSI: Metabolomics standards initiative
MWAS: Metabolome-wide association studies
NGS: Next-generation sequencing
NHST: Null hypothesis significance testing
PCA: Principal component analysis
SNP: Single nucleotide polymorphism
